# Systolic Blood Pressure Variability in Acute Ischemic Stroke: A Predictor of Infarct Growth and Hemorrhagic Transformation

**DOI:** 10.3390/biomedicines13092189

**Published:** 2025-09-07

**Authors:** Oana Elena Sandu, Carina Bogdan, Adrian Apostol, Mihaela Adriana Simu, Lina Haj Ali, Loredana Suhov, Amanda Claudia Schuldesz, Viviana Mihaela Ivan

**Affiliations:** 1 Department VII, Internal Medicine II, Discipline of Cardiology, “Victor Babeş” University of Medicine and Pharmacy, Eftimie Murgu Sq. No. 2, 300041 Timişoara, Romania; oana.ciolpan@umft.ro (O.E.S.); adrian.apostol@umft.ro (A.A.); ivan.viviana@umft.ro (V.M.I.); 2 Doctoral School, “Victor Babeş” University of Medicine and Pharmacy, Eftimie Murgu Sq. No. 2, 300041 Timişoara, Romania; lina.haj-ali@umft.ro (L.H.A.); loredana.ogarcin@umft.ro (L.S.); 3 Department of Neurology, “Victor Babeş” University of Medicine and Pharmacy Timișoara, Eftimie Murgu Sq. No. 2, 300041 Timișoara, Romania; simu.mihaela@umft.ro (M.A.S.); amanda.schuldesz@umft.ro (A.C.S.); 4 Department of Neurology, Clinical Emergency County Hospital “Pius Brînzeu” Timișoara, Bvd. Iosif Bulbuca No. 10, 300736 Timișoara, Romania

**Keywords:** systolic blood pressure variability, medium-term variability, infarct growth, hemorrhagic transformation, acute ischemic stroke, blood pressure monitoring, intravenous thrombolysis

## Abstract

**Background**: Blood pressure variability (BPV) has emerged as an important clinical factor in acute ischemic stroke (AIS), with evidence linking excessive fluctuations in systolic blood pressure (SBP) to secondary brain injury. This study aimed to assess the association between SBP variability during the first week of hospitalization and the risk of early post-stroke complications, specifically hemorrhagic transformation and infarct growth. **Methods**: We conducted a prospective cohort study involving 138 AIS patients admitted to the Pius Brinzeu County Emergency Hospital, Timișoara, between November 2022 and December 2024. Systolic blood pressure (SBP) was assessed three times daily over a period of seven days, with variability determined as the standard deviation (SD) of the recorded values. Patients were categorized based on treatment modality (conservative versus intravenous thrombolysis), and complications were evaluated using repeated computed tomography (CT) imaging. **Results**: SBP variability was significantly higher in patients who developed hemorrhagic transformation (OR 3.64, 95% CI: 2.21–5.99, *p* < 0.001) or infarct growth (OR 1.80, 95% CI: 1.24–2.61, *p* = 0.001). A monotonic trend was observed across SBP variability categories, with complication rates increasing significantly with higher variability levels (*p* < 0.001 for hemorrhagic transformation; *p* = 0.001 for infarct growth). In multivariable models, SBP variability remained an independent predictor of hemorrhagic transformation in both the conservative group (OR 4.78, 95% CI: 2.07–37.14, *p* = 0.02) and thrombolysis group (OR 1.47, 95% CI: 1.13–2.08, *p* = 0.01), and was also associated with infarct growth in the thrombolysis group (OR 1.51, 95% CI: 1.13–2.25, *p* = 0.02). **Conclusions**: Medium-term SBP variability is an independent predictor of early ischemic and hemorrhagic complications following AIS, particularly in patients receiving thrombolysis. These findings support the need for targeted strategies to stabilize BP during the acute phase of stroke care.

## 1. Introduction

Stroke remains a leading cause of death and long-term disability worldwide, accounting for over 12 million new cases and 6.5 million deaths annually, with ischemic stroke comprising approximately 85% of all cases; due to increasing life expectancy, stroke incidence is projected to rise substantially, and cases in adults over 60 are expected to nearly triple by 2050 [1,2]. The growing burden reflects not only demographic trends but also the rising prevalence of modifiable vascular risk factors.

High blood pressure (BP) remains a primary modifiable risk factor in the development of stroke and other cardiovascular events, particularly in cases of lacunar infarction and atherothrombotic stroke sub-types [3,4,5]. Despite advances in prevention and treatment, hypertension continues to pose a significant public health concern [6].

Elevated BP, defined by the World Health Organization as ≥140/90 mmHg, is observed in up to 75% of acute stroke cases [7,8]. Although BP tends to decline within the first week post-onset, nearly 40% of patients remain hypertensive during the subacute phase [9].

Persistent elevation of SBP in AIS is frequently driven by chronic hypertension, suboptimal adherence to antihypertensive treatment, and acute stress responses that occur in the early phase of stroke. Wallace et al. demonstrated that impaired endothelial function contributes substantially to increased arterial stiffness in isolated systolic hypertension and aging [10], a mechanism that also helps explain the elevated SBP observed in AIS patients.

High SBP directly contributes to vascular damage by inducing endothelial dysfunction and promoting arterial stiffness, mechanisms which in turn increase the risk of cerebrovascular injury [11]. Arboix et al. have shown that SBP variability further exacerbates this risk by applying mechanical stress on the arterial walls, accelerating vascular remodeling and atherosclerotic processes; these effects are particularly relevant in ischemic stroke sub-types associated with small-vessel disease and atherothrombosis [5].

Elevated SBP, mean arterial blood pressure (MABP), and diastolic BP (DBP) during the acute phase of stroke are associated with worse clinical outcomes, including mortality and combined endpoints of death and disability [9].

Major cardiovascular comorbidities were considered besides SBP variability, including atrial fibrillation (AF) and heart failure (HF), due to their known impact on stroke outcomes. AF, the most common supraventricular arrhythmia in adults, is characterized by uncoordinated atrial activity and an irregular ventricular response, and frequently coexists with HF [12]. HF itself is a complex clinical syndrome marked by impaired ventricular filling and ejection [13]. The coexistence of AF and HF is clinically significant, as it substantially increases morbidity and mortality, and reduces quality of life, thereby amplifying the risk of poor outcomes following acute ischemic stroke [14].

Evidence suggests that BPV may outperform average SBP as a predictor of all-cause and cardiovascular mortality, as well as stroke and other cardiac events [15,16].

Rothwell et al. demonstrated that SBP variability is a powerful predictor of stroke risk, independent of mean BP levels [17]. These findings support growing evidence that marked BP fluctuations early after stroke onset adversely impact neurological outcomes and suggest that BPV may represent a therapeutic target in both the subacute and chronic phases of ischemic stroke [18].

Emerging clinical data, including findings from recent cohort studies, support SBP variability as a strong predictor of both ischemic and hemorrhagic stroke risk [19,20]. The predictive value of SBP variability is explained by impaired cerebral autoregulation in the acute phase of stroke, which makes fluctuations in blood pressure more directly translate into changes in cerebral perfusion, thereby increasing vulnerability to ischemic and hemorrhagic events [21]. Patients with higher SBP fluctuations tend to experience earlier stroke onset, shorter stroke-free survival, and increased mortality. The association appears particularly pronounced for hemorrhagic events, underscoring the relevance of BPV as a modifiable cerebrovascular risk factor [22].

Rapid surges or drops in blood pressure can trigger adverse events after stroke onset, such as hemorrhagic transformation or worsening ischemia. BPV has been identified as an independent predictor of both short- and long-term outcomes in patients with acute stroke [23].

BPV can be assessed over different timeframes: long term (e.g., seasonal or visit-to-visit changes), medium term (day-to-day fluctuations over several days), and short term (intra-day changes). Regardless of the interval, elevated BPV is associated with a poorer cardiovascular prognosis, even when mean BP levels are controlled [4].

Medium-term BPV refers to fluctuations in systolic blood pressure measured over a period of 3 to 7 consecutive days. It captures day-to-day variability and has demonstrated clinical relevance in predicting early outcomes following acute ischemic stroke [24].

There is ongoing debate regarding thresholds that define excessive BPV. In the ASCOT (Anglo-Scandinavian Cardiac Outcomes Trial), an SD of ≥10 mmHg in SBP was associated with elevated cardiovascular risk. Other studies propose that variability exceeding 12–13 mmHg may indicate clinically significant risk requiring attention [25].

Hemorrhagic transformation, particularly symptomatic intracerebral hemorrhage, remains the most-feared complication of intravenous thrombolysis in AIS. Current consensus recommends maintaining BP below 180/105 mmHg during the first 24 h following thrombolytic therapy, although the optimal BP management strategy beyond this window remains uncertain [26].

Woods et al. reported that elevated BP variability was linked to greater infarct growth, a higher risk of hemorrhagic transformation, and a reduced likelihood of favorable functional recovery following stroke [27].

Our study investigates the prognostic relevance of SBP variability during the first week of hospitalization following AIS, with a specific focus on its association with early complications such as hemorrhagic transformation and infarct growth. By stratifying patients based on treatment type and analyzing outcomes through repeated imaging, our aim is to assess the clinical impact of medium-term SBP variability in both conservatively and thrombolysis-treated patients.

## 2. Materials and Methods

### 2.1. Population

Our prospective study included 138 patients diagnosed with AIS between November 2022 and December 2024. All patients were evaluated at the Emergency Department of the County Emergency Hospital “Pius Brinzeu” in Timisoara, Romania. The diagnosis of AIS was based on clinical presentation and confirmed through CT imaging. All CT scans were performed using General Electric Revolution EVO and General Electric Revolution Ascend systems, both equipped with 128-slice capabilities.

Patients who presented within four and a half hours of symptom onset and had no contraindications received intravenous thrombolytic therapy. Those who arrived outside the thrombolysis time window or had contraindications received standard conservative medical management. All patients were monitored for a period of seven days during hospitalization in the Neurology Department. All patients underwent non-contrast CT imaging during the first 7 days of hospitalization to assess for hemorrhagic transformation and infarct growth. The initial CT scan used for comparison was the baseline non-contrast CT performed at admission (“time zero”). In cases where the admission CT did not demonstrate a visible infarct, the subsequent CT obtained 24 h after presentation was considered the reference scan. Follow-up CT imaging was performed during the first seven days after the reference scan, according to clinical indication rather than at predetermined time intervals. The infarct size was measured on non-contrast CT scans as the maximum linear dimensions of the hypodense lesion in the axial plane. Two perpendicular diameters (length and width) were recorded. Measurements were performed manually using the 3dnet (Biotronics3D, London, UK). Infarct size was independently assessed by two experienced radiologists in order to reduce interobserver variability and minimize measurement error.

Patients were excluded from the study if they underwent mechanical thrombectomy. Additional exclusion criteria included lack of informed consent and insufficient clinical or imaging data for analysis.

All patients received antihypertensive treatment in accordance with current European Society of Hypertension and European Society of Cardiology guidelines, along with appropriate pharmacological management of relevant comorbidities.

Informed consent was obtained from each participant prior to inclusion, in accordance with the ethical standards of the Declaration of Helsinki and the European Union General Data Protection Regulation (GDPR). The study protocol was approved by the institutional ethics committee.

### 2.2. Blood Pressure Measurement

SBP was measured with the patient in a supine position using an automated oscillometric device (OMRON M3 Comfort) applied to the non-paretic upper arm. Cuff size was selected according to the measured mid-arm circumference to ensure proper fit. The cuff was positioned on the upper arm, approximately 2–3 cm above the antecubital fossa, following standard clinical guidelines.

At each measurement session, three consecutive SBP measurements were obtained; each SBP measurement was separated by a 2-min rest interval. The mean of these three values was calculated and recorded as the representative SBP mean for that measurement session. A measurement session was defined as any of the three daily scheduled blood pressure assessments: morning (08:00–10:00), midday (12:00–14:00), and evening (18:00–20:00). Each measurement session was performed after antihypertensive medication administration, at consistent intervals throughout the seven-day observation period. All SBP measurement values and calculated SBP means were systematically recorded at the time of acquisition during the respective measurement session.

### 2.3. Systolic Blood Pressure Variability

SBP variability was defined as the degree of fluctuation in SBP means over time. For each patient, SBP variability was quantified as the SD of all SBP means recorded across the seven-day observation period. This measure reflects the dispersion of daily SBP means around the patient-specific average SBP, providing an index of blood pressure stability. Patient-specific average SBP was defined as the arithmetic mean of all SBP means recorded during the seven-day observation period for a respective patient. Higher SD values indicate greater variability and less-consistent BP control.

For subsequent analysis, SBP variability was categorized into four clinically relevant ordinal groups: <10 mmHg (low), 10–14 mmHg (medium), 14–20 mmHg (high), and ≥20 mmHg (very high).

By focusing on medium-term variability during the acute post-stroke phase, the study aimed to assess whether short-term fluctuations in SBP are predictive of adverse events such as infarct growth or hemorrhagic transformation.

### 2.4. Statistical Analysis

We employed descriptive statistics to sum up the demographic and clinical traits of the people in the research. Continuous variables, including age and SBP variability, were presented as means with SD, while categorical variables (e.g., sex, smoking status, and comorbidities) were expressed as percentages.

To assess the distribution of SBP variability, normality was tested using the Shapiro–Wilk and Anderson–Darling tests. We applied the Anderson–Darling test in order to improve sensitivity, especially for finding changes in the distribution tails. Distributional assumptions were further evaluated visually to assess normality via quantile–quantile (Q–Q) plots, where observed SBP variability values were displayed on the vertical axis against theoretical quantiles of a standard normal distribution on the horizontal axis, with systematic deviations from the reference line interpreted as evidence of non-normality.

The Wilcoxon rank-sum test was applied to compare SBP variability between patients with and without complications (hemorrhagic transformation and infarct growth). This non-parametric approach was chosen because it does not assume normality and is robust to skewed or heavy-tailed distributions. This test was repeated for both the entire cohort and treatment-defined subgroups (conservatively treated and thrombolysis-treated patients), to assess whether distributional patterns and outcome associations differed by intervention type.

For trend analysis, SBP variability was categorized as previously specified. To evaluate whether increasing SBP variability was associated with a progressive increase in complication rates, the Cochran–Armitage trend test was employed. This non-parametric test was selected for its suitability in detecting monotonic trends between an ordinal predictor and a binary outcome.

To complement this non-parametric analysis and to quantify the magnitude of association, logistic regression models were also fitted using the numeric form of the SBP variability categories. This allowed estimation of the linear trend in complication risk (expressed as odds ratios) across the SBP variability spectrum. The regression coefficient represented the change in log odds of outcome per category increase in SBP variability. Models were fitted separately for each outcome and subgroup.

Finally, to identify independent predictors of hemorrhagic transformation and infarct growth, multivariate logistic regression analyses were performed within each treatment group. Predictor variables were selected based on clinical relevance and included SBP variability, age, sex, and vascular risk factors.

All statistical procedures were conducted using RStudio Version 2025.05.1 and R Version 4.5.1. Statistical significance was considered for *p* < 0.05.

Approval of the study protocol was provided by the ethical committee of our institution.

## 3. Results

### 3.1. Study Population Description

The mean age of the study cohort was 69.57 years (SD ± 9.68). Of the 138 participants, 55.80% were male, and 44.20% were female. The most prevalent comorbid condition was arterial hypertension, affecting 75.36% of the subjects, followed by dyslipidemia with 73.91% and diabetes mellitus with 50.72%.

Atrial fibrillation was observed in 41.30% of patients, while obesity and heart failure were present in 42.03% and 35.51%, respectively. Additionally, coronary artery disease was documented in 28.99% of cases, and chronic kidney disease was reported in 18.84%. Regarding lifestyle-related risk factors, 40.58% of the participants were current or former smokers, and 18.12% reported regular alcohol consumption (Table 1).

### 3.2. SBP Variability Analysis for Entire Study Population

**Figure 1:**
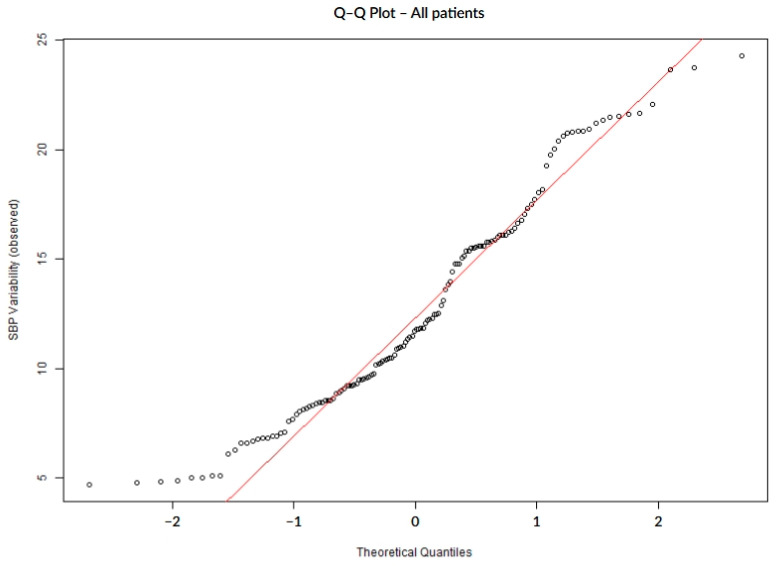
Q–Q plot illustrating the deviation from normality in SBP variability across all patients.

**Figure 2:**
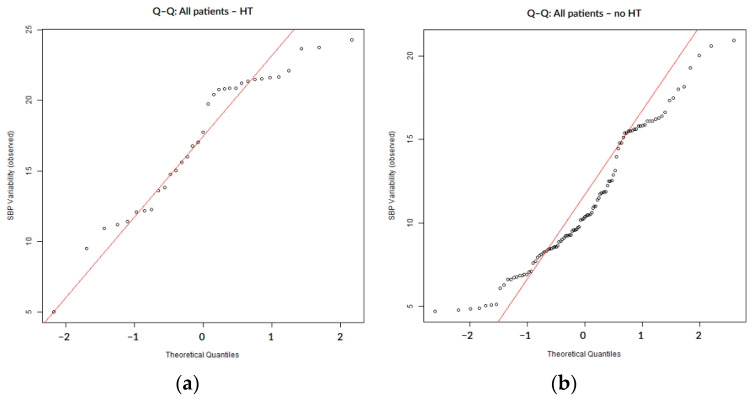
Q–Q plots of SBP variability stratified by the presence (**a**) or absence (**b**) of hemorrhagic transformation.

**Figure 3:**
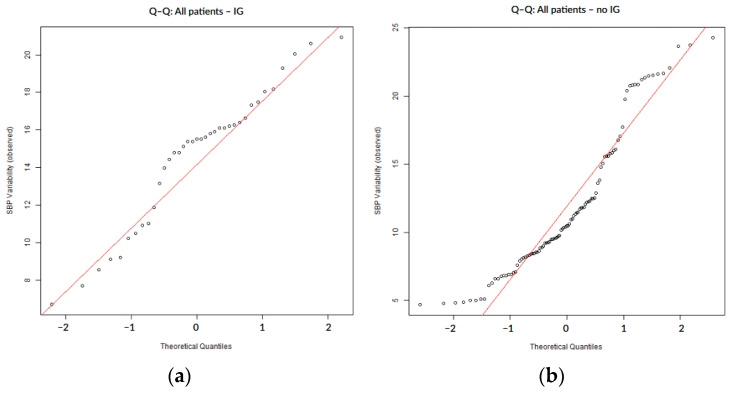
Q–Q plots of SBP variability stratified by the presence (**a**) or absence (**b**) of infarct growth.

SBP variability during the first 7 days of hospitalization did not follow a normal distribution, as confirmed by both numerical (Table 2) and visual assessments. The Q–Q plots showed systematic deviations from the expected diagonal line, particularly in the lower and upper tails. These distributional characteristics were consistently observed in the overall cohort (Figure 1) as well as within subgroups defined by the presence or absence of hemorrhagic transformation (Figure 2) and infarct growth (Figure 3).

When comparing SBP variability between patients who developed hemorrhagic transformation and those who did not, a significantly higher degree of variability was observed in the complication group (Table 3). A similar trend was noted for patients who experienced infarct growth, who also exhibited greater SBP fluctuations compared to those without this outcome.

### 3.3. SBP Variability Analysis for Conservatively Treated Patients

**Figure 4:**
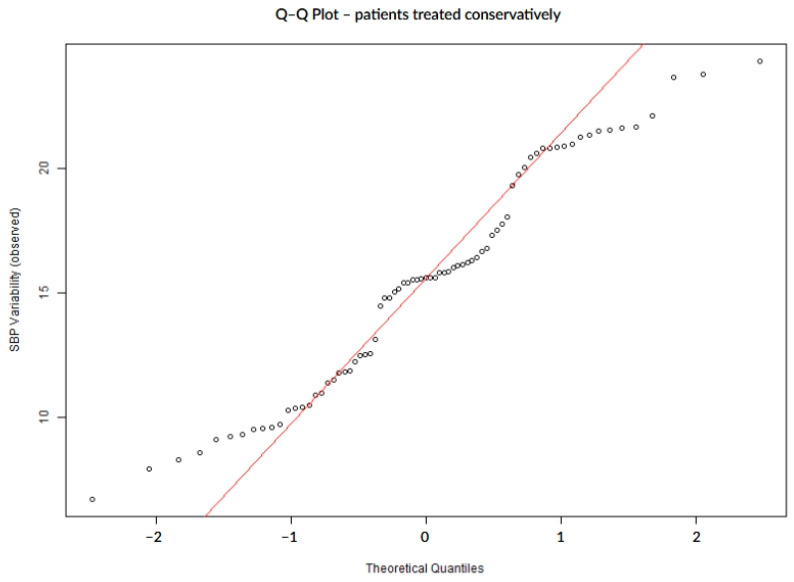
Q–Q plot of SBP variability in conservatively treated patients.

**Figure 5:**
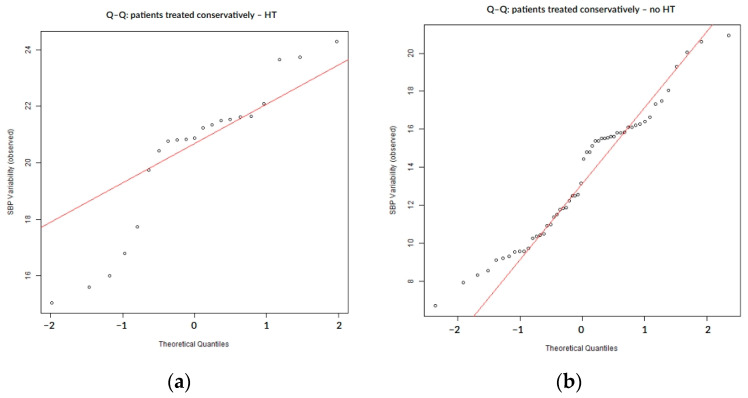
Q–Q plots of SBP variability stratified by hemorrhagic transformation status: (**a**) patients treated conservatively with hemorrhagic transformation; (**b**) patients treated conservatively without hemorrhagic transformation; and HT = hemorrhagic transformation.

**Figure 6:**
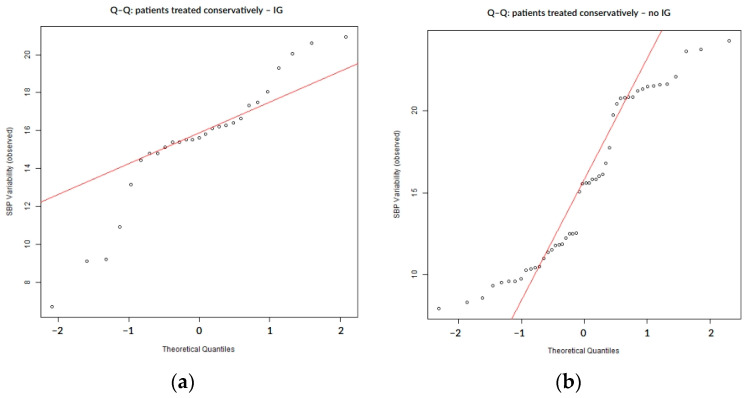
Q–Q plots of SBP variability stratified by infarct growth status: (**a**) patients treated conservatively with infarct growth; (**b**) patients treated conservatively without infarct growth; and IG = infarct growth.

In the subgroup of patients managed conservatively (n = 75), the distribution of SBP variability again deviated from normality, as shown by both statistical and graphical assessments (Table 4, Figure 4). Although less pronounced than in the overall cohort, Q–Q plots revealed moderate deviation from the theoretical normal line, particularly in the upper tail (Figure 4). These deviations were also visible in patients with and without hemorrhagic transformation or infarct growth (Figure 5 and Figure 6).

Among conservatively treated patients, SBP variability was significantly higher in those who developed hemorrhagic transformation (*p* < 0.001) compared to those who did not (*p* = 0.9). In contrast, no relevant difference in SBP variability was observed between patients with and without infarct growth (Table 5).

### 3.4. SBP Variability Analysis for Patients Treated by IVT

**Figure 7:**
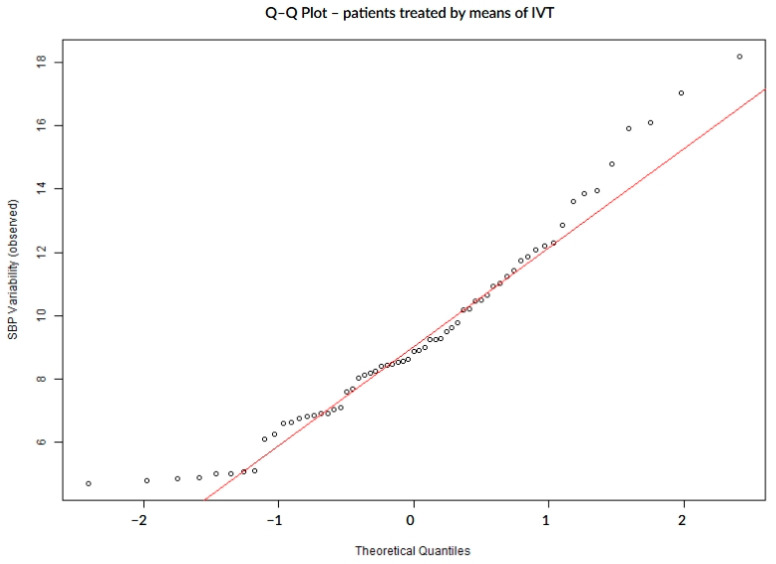
Q–Q plot of SBP variability in patients treated with intravenous thrombolysis.

**Figure 8:**
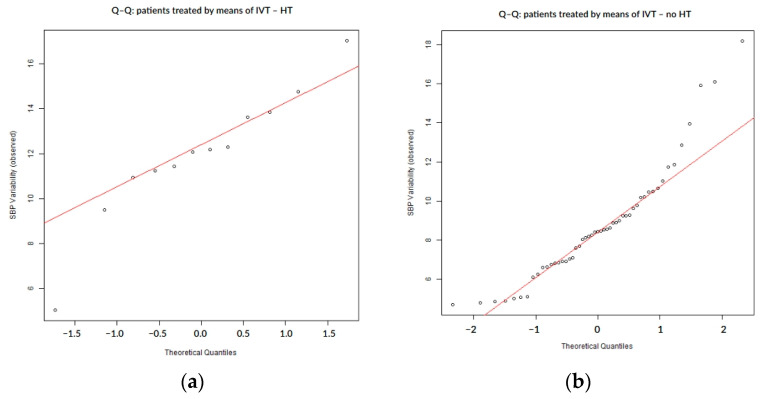
Q–Q plots of SBP variability stratified by hemorrhagic transformation status in IVT-treated patients: (**a**) IVT-treated patients with hemorrhagic transformation; (**b**) IVT-treated patients without hemorrhagic transformation; and HT = hemorrhagic transformation.

**Figure 9:**
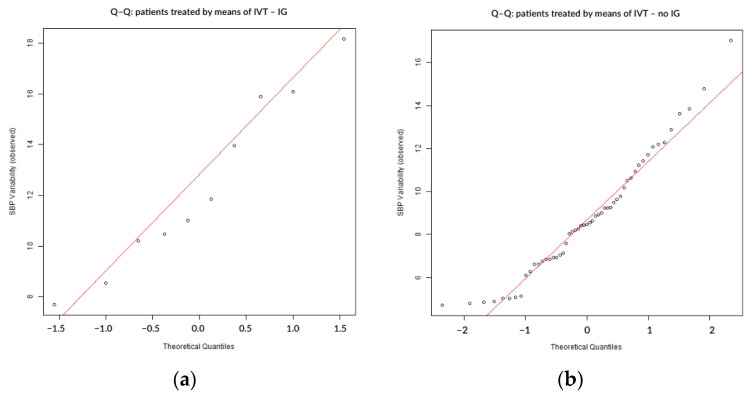
Q–Q plots of SBP variability stratified by infarct growth status in IVT-treated patients: (**a**) IVT-treated patients with infarct growth; (**b**) IVT-treated patients without infarct growth; and IG = infarct growth.

In patients treated with intravenous thrombolysis (IVT, n = 63), SBP variability demonstrated mild deviations from normality, as indicated by both statistical tests and Q–Q plot assessment (Table 6, Figure 7). While the overall distribution was closer to normal compared to other subgroups, the presence of asymmetry and tail deviations still suggested non-normal behavior, particularly in patients who experienced post-stroke complications (Figure 8 and Figure 9).

Within this subgroup, SBP variability was significantly higher among patients who developed hemorrhagic transformation (*p* < 0.001) or infarct growth (*p* < 0.01) compared to those without these complications. These findings support the hypothesis that medium-term fluctuations in SBP are more strongly associated with adverse outcomes in patients receiving reperfusion therapy (Table 7).

### 3.5. Association Between SBP Variability and Post-Stroke Complications

In the entire study population, analyzed independently of treatment strategy, increasing categories of SBP variability were associated with progressively higher rates of both hemorrhagic transformation (Z = −5.88, *p* < 0.001) and infarct growth (Z = −3.20, *p* = 0.001), indicating a strong and consistent upward trend (Table 8).

Among patients managed conservatively, a similar association was observed for hemorrhagic transformation (Z = −5.38, *p* < 0.001), but no trend was evident for infarct growth (Z = −0.54, *p* = 0.590), suggesting limited predictive relevance in this subgroup.

In contrast, patients treated with intravenous thrombolysis exhibited clear trends for both complications. Higher SBP variability categories were linked to increased rates of hemorrhagic transformation (Z = −3.36, *p* < 0.001) and infarct growth (Z = −3.55, *p* < 0.001), supporting the hypothesis that SBP variability contributes to poorer outcomes in the setting of reperfusion therapy.

The findings in Table 9 further confirm the association between SBP variability and adverse outcomes. In the overall cohort, each increase in SBP variability category was associated with significantly higher odds of both hemorrhagic transformation (OR 3.64, 95% CI: 2.21–5.99, *p* < 0.001) and infarct growth (OR 1.80, 95% CI: 1.24–2.61, *p* = 0.002).

In the conservatively treated group, SBP variability remained a strong predictor of hemorrhagic transformation (OR 23.02, 95% CI: 5.50–96.34, *p* < 0.001), while no significant association was observed for infarct growth (OR 1.14, 95% CI: 0.70–1.85, *p* = 0.591).

Among patients who received thrombolytic therapy, SBP variability was significantly associated with an increased risk of both hemorrhagic transformation (OR 4.68, 95% CI: 1.69–12.97, *p* = 0.003) and infarct growth (OR 5.58, 95% CI: 1.84–16.95, *p* = 0.002).

### 3.6. Independent Predictors of Hemorrhagic Transformation and Infarct Growth

A multivariable modeling approach was applied within each treatment group in order to identify independent predictors of hemorrhagic transformation and infarct growth. Clinically relevant variables were considered, including SBP variability, age, sex, and vascular risk factors (Table 10).

In the conservatively treated group, elevated SBP variability was independently associated with a significantly increased risk of hemorrhagic transformation (OR = 4.78, 95% CI: 2.07–37.14, *p* = 0.02). No variables, including SBP variability, were significantly associated with infarct growth in this group.

Among patients who received IVT, SBP variability emerged as a significant predictor for both infarct growth and hemorrhagic transformation. Each increase in SBP variability was associated with higher odds of hemorrhagic transformation (OR = 1.47, 95% CI: 1.13–2.08, *p* = 0.01) and infarct growth (OR = 1.51, 95% CI: 1.13–2.25, *p* = 0.02).

Other factors, including age, sex, heart failure, hypertension, atrial fibrillation, coronary artery disease, diabetes mellitus, chronic kidney disease, obesity, dyslipidemia, alcohol use, and smoking status, were included in the models but did not demonstrate a significant independent association with either outcome in any treatment subgroup.

## 4. Discussion

Our research shows that SBP variability during the first week of hospitalization is a strong predictor of early post-stroke complications in patients with AIS. Higher SBP variability was significantly associated with both infarct growth and hemorrhagic transformation, with the association being particularly pronounced in patients who received thrombolytic therapy. These results highlight the prognostic relevance of medium-term SBP fluctuations during the acute phase and suggest that higher variability may contribute to increased vulnerability to secondary cerebral injury. The observed relationship underscores the need for more-refined blood pressure management strategies in the early stages of AIS to mitigate the risk of adverse outcomes, findings that are consistent with a recent study by Liao et al., who examined stroke care dynamics and early in-hospital complications [28].

The average age of our study population was 69.57 years (SD ± 9.68), which is in line with previous findings that emphasize the strong association between advancing age and AIS. In the cohort analyzed by Li et al., the average age was reported as 73.6 years, reflecting a similarly aged stroke population [29]. Age is widely recognized as the most significant non-modifiable risk factor for stroke, with incidence rates doubling every decade after the age of 55, as noted by Yousufuddin and Young [30]. Notably, approximately 75% of all strokes occur in individuals aged 65 or older, underscoring the heightened cerebrovascular vulnerability in older adults. Furthermore, research by Edrissi et al. indicates increased clinical severity in AIS patients aged 65–74 and those ≥75 years, particularly in the presence of comorbid conditions such as heart failure [31].

In our cohort, a high prevalence of cardiovascular and metabolic risk factors was observed. For instance, heart failure (present in 35.51% of our patients) has been identified as a strong determinant of stroke severity and poor functional outcomes, largely due to impaired cardiac output and cerebral perfusion, contributing to both ischemic injury and hemorrhagic risk [32]. Similarly, atrial fibrillation, documented in 41.30% of our cohort, represents a key etiological factor for cardioembolic stroke and is associated with larger infarct volumes and a higher recurrence risk [33].

Jiang et al. highlighted the importance of lipid metabolic dysregulation in intracerebral hemorrhage by linking cerebrospinal fluid lipid profiles with early- and late-phase hemorrhagic injury [34]. These findings point to a systemic vulnerability that may interact with hemodynamic instability. Similarly, Zhang et al. demonstrated in a rodent model that ischemia/reperfusion injury results in lipid metabolic disturbance that contributes to cognitive dysfunction and may modulate infarct progression [35]. While their findings are preclinical, they suggest that vascular metabolic stress, compounded by SBP variability, may further compromise cerebral integrity [34,35].

Diabetes mellitus, affecting over half of our patients (50.72%), is a well-established contributor to stroke risk through mechanisms involving endothelial dysfunction, accelerated atherosclerosis, and slower neurovascular repair. Diabetic patients also tend to have worse recovery outcomes, as noted in recent research, highlighting an increased stroke incidence and complications in this population [36]. Chronic kidney disease (CKD), present in 18.84% of the study group, further exacerbates vascular fragility and inflammation, potentially amplifying the risk of both ischemic extension and hemorrhagic transformation [37].

Other modifiable risk factors such as smoking (40.58%), alcohol use (18.12%), obesity (42.03%), and dyslipidemia (73.91%) were also highly prevalent in our cohort and may interact to increase vascular instability and stroke complications [38,39,40].

Consistent with our findings, Woods et al. have demonstrated that increased blood pressure variability is independently associated with a higher risk of infarct growth and parenchymal hemorrhage, supporting the notion that fluctuations in systolic blood pressure may exacerbate secondary brain injury [27].

Stratified analyses further revealed that patients with greater BP variability had a higher incidence of hemorrhagic transformation [41].

In a study by Liu et al., they demonstrated that in the acute phase following IVT, elevated SBP variability within the first 6 h was correlated strongly with the development of parenchymal hematoma and symptomatic intracerebral hemorrhage (sICH); moreover, SBP fluctuations recorded across all time windows in the first 24 h post-treatment were significant predictors of long-term outcomes [26].

Similarly, Kim et al., in a cohort undergoing successful endovascular reperfusion therapy, showed that temporal SBP variability was independently associated with the occurrence of sICH, even after adjusting for clinical confounders [42].

We found that SBP variability, assessed over the first seven days of hospitalization, indicates a strong association with HT among patients with AIS. In our cohort, higher SBP variability was consistently associated with increased odds of HT, particularly in both conservatively treated and thrombolysis-treated subgroups. This is supported by previous findings which indicated that blood pressure variability independently contributes to the development of parenchymal hemorrhage and hemorrhagic complications in the acute phase of stroke [21]. Similarly, Woods et al. reported a strong relationship between elevated SBP variability and increased risk of hemorrhagic transformation [27]. Moreover, a study by Ko et al. emphasized that BP variability, regardless of treatment strategy, remained a key factor in HT occurrence, reinforcing the need to consider variability rather than solely BP thresholds in post-stroke care [41].

Infarct growth was also significantly linked to SBP variability in our overall population and in the thrombolysis-treated subgroup. These findings are in agreement with prior studies highlighting that elevated SBP variability is associated with infarct expansion, which in turn contributes to worsened functional outcomes [27]. Notably, evidence from Chung et al. demonstrated that greater BP variability correlates with increased infarct volume and neurological deterioration in the early post-stroke phase, further emphasizing the pathophysiological relevance of fluctuating SBP levels [43].

The prognostic value of SBP variability appears to be consistent regardless of treatment strategy. Through our results, we found no significant interaction between treatment type and the effect of SBP variability on the risk of complications. This mirrors the results reported by Ko et al., where the effects of BP variability on HT did not differ significantly by thrombolysis status [41]. In contrast, some studies have failed to demonstrate a predictive value of BPV for hemorrhagic complications following thrombolysis [44]. However, our results reinforce the broader perspective presented by Endo et al., which underscores the importance of blood pressure fluctuations as a reliable predictor of early stroke outcomes, independent of mean BP levels [45].

Our findings reinforce the clinical relevance of medium-term SBP variability as a robust predictor of both hemorrhagic and ischemic complications. The consistency of these results across multiple analytic methods and subgroups supports SBP variability as an important and potentially modifiable parameter in acute stroke management.

We acknowledge several limitations inherent to our study. First, the study was conducted at a single center, which may limit external validity and applicability to other clinical settings. Second, the modest sample size (n = 138) may reduce statistical power and restrict the robustness of subgroup analyses, particularly when stratifying by treatment type or complication outcome. Third, the cohort predominantly consisted of an elderly population (mean age: 69.57 years), which may limit the generalizability of our findings to younger individuals. Fourth, follow-up CT imaging was performed during the first seven days after the reference scan, based on clinical indication rather than at fixed time intervals. Lastly, while SBP variability was carefully measured over a seven-day period, the specific classes, dosages, and timing of antihypertensive medications were not recorded, potentially confounding the observed associations.

Future research should aim to clarify the prognostic value of medium-term SBP variability in predicting long-term functional outcomes, particularly at 30, 60, and 90 days following acute ischemic stroke. Such investigations would provide valuable insight into whether early fluctuations in SBP carry implications beyond the immediate hospitalization period. Additionally, SBP variability should be further explored as a potential therapeutic target in acute stroke management, assessing whether specific strategies aimed at reducing variability, rather than solely lowering absolute BP values, can translate into improved clinical outcomes. Ongoing trials such as BEST-II are exploring whether tighter blood pressure control or modulation of BP dynamics following reperfusion therapy can improve clinical outcomes and reduce secondary complications, including hemorrhagic transformation [46]. It is also important to investigate the time-dependent effects of BPV, distinguishing between early fluctuations in the first 24 h and those occurring between days 3 and 7 post-stroke. This approach may help identify critical periods during which blood pressure stabilization efforts could be most beneficial in mitigating ischemic or hemorrhagic complications.

## 5. Conclusions

Our study underscores the prognostic value of SBP variability during the first seven days of hospitalization as an independent predictor of post-stroke complications in patients with AIS. Higher SBP variability was significantly associated with an increased risk of both hemorrhagic transformation and infarct growth, with this relationship particularly evident in patients treated with intravenous thrombolysis.

A monotonic trend was observed across SBP variability categories, with complication rates rising in parallel with increasing variability. Multivariable analyses confirmed SBP variability as a strong and independent predictor of hemorrhagic transformation in both conservatively and IVT-treated subgroups, while its association with infarct growth was significant only in thrombolysis recipients.

These findings reinforce the importance of monitoring not only absolute SBP values but also their variability in the early post-stroke period. SBP variability may serve as a useful clinical indicator, with the potential to guide more-personalized blood pressure management strategies aimed at minimizing secondary brain injury.

Future investigations should further assess the long-term prognostic value of medium-term SBP variability and explore whether targeted interventions to reduce variability could improve functional outcomes beyond the acute phase of stroke care.

## Figures and Tables

**Table 1:** Study population description.

Characteristics	Values, N = 138 (%)
Age	69.57 (9.68) *
Sex	M: 55.80 F: 44.20
Heart failure	35.51
High blood pressure	75.36
Atrial fibrillation	41.30
Coronary artery disease	28.99
Diabetes mellitus	50.72
Chronic kidney disease	18.84
Obesity	42.03
Dyslipidemia	73.91
Alcoholic	18.12
Smoker	40.58

* Age expressed in mean value and standard deviation in the form of mean (SD).

**Table 2:** SBP variability normality test.

Group	*p*
Entire study population	<0.001
Entire study population	<0.001

Note: The first row reports the *p*-value for the Shapiro–Wilk test; the second row reports the *p*-value for the Anderson–Darling test.

**Table 3:** Comparison of SBP variability between patients with and without post-stroke complications (hemorrhagic transformation and infarct growth).

Group	W	Post-Stroke Complications	*p*
HT	589.5	33	<0.001
IG	1185.5	37	0.001

HT = hemorrhagic transformation; IG = infarct growth.

**Table 4:** Normality testing results for SBP variability in the conservative treatment group.

Group	*p*
Entire study population	0.02
Entire study population	0.01

Note: The first row reports the *p*-value for the Shapiro–Wilk test; the second row reports the *p*-value for the Anderson–Darling test.

**Table 5:** Comparison of SBP variability by complication status (hemorrhagic transformation and infarct growth) in conservatively treated patients.

Group	W	Post-Stroke Complications	*p*
HT	75.5	21	<0.001
IG	643	27	0.9

HT = hemorrhagic transformation; IG = infarct growth.

**Table 6:** Normality testing results for SBP variability in the thrombolysis-treated group.

Group	*p*
Entire study population	0.01
Entire study population	0.05

Note: The first row reports the *p*-value for the Shapiro–Wilk test; the second row reports the *p*-value for the Anderson–Darling test.

**Table 7:** Comparison of SBP variability by outcome (hemorrhagic transformation and infarct growth) in patients treated with IVT.

Group	W	Post-Stroke Complications	*p*
HT	110	12	<0.001
IG	110	10	<0.01

HT = hemorrhagic transformation; IG = infarct growth.

**Table 8:** Trend across SBP variability categories.

Group	Outcome	Z	*p*
Entire study population (n = 138)	HT	−5.88	<0.001
	IG	−3.20	0.001
Conservative (n = 75)	HT	−5.38	<0.001
	IG	−0.54	0.590
IVT (n = 63)	HT	−3.36	<0.001
	IG	−3.55	<0.001

HT = hemorrhagic transformation; IG = infarct growth; and IVT = intravenous thrombolysis.

**Table 9:** Association between SBP variability and complications.

Group	Outcome	Estimate	Error	Z	*p*	OR (95% CI)
Entire study population (n = 138)	HT	1.293	0.254	5.090	<0.001	3.64 (2.21–5.99)
	IG	0.586	0.189	3.100	0.002	1.80 (1.24–2.61)
Conservative (n = 75)	HT	3.137	0.730	4.290	<0.001	23.02 (5.50–96.34)
	IG	0.133	0.247	0.540	0.591	1.14 (0.70–1.85)
IVT (n = 63)	HT	1.542	0.521	2.960	0.003	4.68 (1.69–12.97)
	IG	1.720	0.567	3.040	0.002	5.58 (1.84–16.95)

OR = odds ratio; CI = confidence interval; HT = hemorrhagic transformation; IG = infarct growth; and IVT = intravenous thrombolysis.

**Table 10:** Analysis of predictors for hemorrhagic transformation and infarct growth, stratified by treatment subgroups.

	Conservative Treatment (N = 75)				IVT (N = 63)			
	HT		IG		HT		IG	
Characteristics	OR (95% CI)	*p*	OR (95% CI)	*p*	OR (95% CI)	*p*	OR (95% CI)	*p*
SBP variability	4.78 (2.07–37.14)	0.02	0.93 (0.79–1.10)	0.41	1.47 (1.13–2.08)	0.01	1.51 (1.13–2.25)	0.02
Age	0.87 (0.63–1.02)	0.23	0.99 (0.94–1.04)	0.63	1.05 (0.94–1.21)	0.39	1.05 (0.92–1.24)	0.50
Sex	0.33 (0.02–4.07)	0.40	1.64 (0.57–4.90)	0.37	0.38 (0.04–2.53)	0.35	0.65 (0.08–4.64)	0.67
Heart failure	2.23 (0.05–95.06)	0.67	1.56 (0.40–6.32)	0.52	3.24 (0.35–32.52)	0.29	0.87 (0.07–8.05)	0.91
High blood pressure	0.06 (0.00–50.13)	0.39	2.96 (0.54–24.10)	0.25	0.30 (0.03–2.08)	0.25	4.03 (0.53–49.00)	0.21
Atrial fibrillation	0.06 (0.00–1.32)	0.16	1.10 (0.35–3.50)	0.87	0.74 (0.10–4.89)	0.76	2.15 (0.30–18.40)	0.45
Coronary artery disease	1.44 (0.04–68.88)	0.85	1.11 (0.31–3.90)	0.87	0.33 (0.03–2.93)	0.34	4.35 (0.47–50.46)	0.20
Diabetes mellitus	1.72 (0.08–38.56)	0.72	0.71 (0.22–2.28)	0.56	1.20 (0.18–8.35)	0.84	0.35 (0.04–2.61)	0.33
Chronic kidney disease	0.17 (0.00–3.10)	0.28	0.87 (0.21–3.45)	0.85	7.60 (0.76–98.44)	0.09	0.32 (0.01–6.08)	0.47
Obesity	15.44 (0.97–1446.90)	0.13	0.54 (0.15–1.81)	0.32	0.73 (0.11–4.28)	0.73	1.34 (0.15–12.49)	0.78
Dyslipidemia	0.03 (0.00–0.65)	0.09	2.03 (0.50–8.89)	0.33	2.96 (0.31–49.93)	0.38	1.37 (0.13–16.59)	0.79
Alcoholic	0.68 (0.02–18.44)	0.81	1.32 (0.32–5.17)	0.69	0.10 (0.00–1.73)	0.18	0.77 (0.03–9.73)	0.85
Smoker	1.78 (0.18–22.27)	0.62	0.72 (0.24–2.06)	0.54	0.24 (0.02–1.61)	0.18	0.82 (0.07–6.50)	0.86

IVT = intravenous thrombolysis; HT = hemorrhagic transformation; IG = infarct growth; OR = odds ratio; CI = confidence interval; and SBP = systolic blood pressure.

## Data Availability

Data is contained within the article.
